# Dialogue matters. Exploring Deaf people’s research experiences in Poland

**DOI:** 10.1093/jdsade/enag006

**Published:** 2026-02-23

**Authors:** Tomasz Krawczyk, Jan Piasecki, Rafał Bołdys, Barbara Butkiewicz, Judyta Koper, Bogusława Kwoka, Franciszek Kwoka, Karolina Rzadek, Marcin Waligora

**Affiliations:** Department of Bioethics and Health Psychology, Faculty of Health Sciences, Jagiellonian University Medical College, Michalowskiego 12, 31-126 Krakow, Poland; Doctoral School of Medical and Health Sciences, Jagiellonian University Medical College, Sw. Lazarza 16, 31-530 Krakow, Poland; Department of Bioethics and Health Psychology, Faculty of Health Sciences, Jagiellonian University Medical College, Michalowskiego 12, 31-126 Krakow, Poland; independent researcher; independent researcher; Office for Persons with Disabilities, Krakow University of Economics, Rakowicka 27, 31-510 Krakow, Poland; independent researcher; independent researcher; independent researcher; Department of Bioethics and Health Psychology, Faculty of Health Sciences, Jagiellonian University Medical College, Michalowskiego 12, 31-126 Krakow, Poland

## Abstract

The meaningful engagement of Deaf people in research poses ethical challenges, yet Deaf people are systematically underrepresented in discussions on ethical research conduct. Our study explores experiences and opinions of Polish Deaf people about research through a bilingual open-ended online survey. We gathered 52 responses and analysed them in collaboration with a Deaf advisory group using an experiential approach to reflexive thematic analysis. We developed six themes, reflecting the challenges, needs and aspirations of Deaf people, embraced under one overarching theme: *Dialogue matters*. Our study highlights social and epistemic inequalities faced by Deaf people in research, as well as the need for greater accessibility and trust. We discuss how the Polish context both reflects and differs from the experiences of Deaf people from other countries. The findings may inform evidence-based recommendations for scientific cooperation between Deaf and hearing people.

Research experiences of Deaf people are rarely the main interest of researchers. Deaf people have historically been—and still are—excluded from participation in scientific debate. Even in the research areas related to deafness, like education of Deaf children, they continue to rarely be active contributors (Benedict & Sass-Lehrer, 2007; Wilson & Winiarczyk, 2014). For example, educational experiences of Deaf students are often analysed from the perspectives of their hearing educators, parents, and peers (Rohatyn-Martin et al., 2023). Despite the increasing popularity of qualitative and co-creation research involving marginalized communities and *people with lived experiences*—including Deaf people—there is little evidence grounded in research experience of Deaf people, particularly concerning their needs and expectations about research participation, engagement and accessibility (Cawthon & Garberoglio, 2021).

Research engagement, both as participants and co-researchers, can be a difficult experience for Deaf people (Singleton et al., 2014). This is especially the case for Deaf sign language users who identify as a sociocultural minority organized around visual–spatial sign languages (Marganiec, 2024). Research and academia, in turn, are the contexts organized around phonocentric norms where hearing ability is assumed and the majority of personnel is hearing, even in the field of *Deaf studies* (Listman & Dingus-Eason, 2018; Talipska, 2019). Moreover, hearing people usually share the medical model of deafness, which frames it as a disability, and tend to hold ableist stereotypes related to deafness (Cue, 2024; Skyer & Cochell, 2020). They also lack cultural competences and awareness of Deaf culture, which may hinder mutual understanding and collaboration (Benedict & Sass-Lehrer, 2007; Talipska, 2024). In such context, research becomes a field of dispute about deafness. There is a clash between the mainstream scientific paradigm (the medical model), and Deaf epistemology (the *Deaf studies* model) which recognizes deafness as a socio-cultural phenomenon and centre the embodied experiences of deafness (Cue et al., 2019; Tomaszewski & Moroń, 2018b). Furthermore, the recognition of the *Deaf studies* model varies between different regions, countries and disciplines (Podgórska-Jachnik, 2013; Tomaszewski & Krzysztofiak, 2025). This complex situation causes ethical and epistemic challenges related to the engagement of Deaf people in research, as well as practical difficulties in designing inclusive research projects.

While Deaf people share many commonalities worldwide, there is also a need to explore local contexts. Deaf people are diverse in regard to the history and scale of faced marginalisation (Wilson & Winiarczyk, 2014; Wright, 2021). This influences both individual and collective awareness of Deaf culture and sociocultural identity, while also shaping social life, legal frameworks, and the provision of accessibility measures (Talipska, 2019; Tomaszewski et al., 2018). For example, in Poland research on Polish Sign Language (*Polski Język Migowy—*PJM) began around the turn of 20^th^ and 21^st^ centuries, followed by the rapid adoption of the American model of Deaf culture among Polish Deaf community (Zdrodowska, 2019). However, this model entered a different, post-communist socio-institutional context, and the shape of Polish Deaf sociocultural identity still lacks in-depth analysis (Tomaszewski & Krzysztofiak, 2025; Zdrodowska, 2019). Thus, inclusion of Deaf perspectives from a variety of regions and settings to the research discourse is crucial for broadening mutual understanding and ensuring ethical research engagement processes.

Research activities are frequently not adapted to the needs of Deaf people (Anderson et al., 2020; Talipska, 2024). Moreover, there is a paucity of research exploring experiences of Deaf people from research, especially experiences of the Deaf sign language users. Also, little research explores perspectives from non-Western communities, as most studies come from the US and the UK (Krawczyk et al., 2024). Thus, we aimed to provide space for Deaf people from Polish communities to express their research experiences. The specific aims of our study were following:

- To explore the experiences, needs, and attitudes of Deaf people related to the research participation and engagement;
- To investigate the accessibility of the research process, relations between Deaf people and researchers, as well as the research impact on Deaf communities;
- To outline the ethical issues present within the research with Deaf people.

## Positionality

Our main research team comprised of three hearing researchers with interdisciplinary backgrounds. TK is a PhD student and trained psychologist whose work for the past five years has focused on disability and Deaf studies. He is communicative in PJM and has professional and personal relations with Deaf community members in Poland. MW and JP are established researchers, trained as philosophers and bioethicists. Both are TK’s supervisors. We are experienced in the conduct of social science research and systematic reviews.

As we are hearing researchers from hearing families, we want to recognize our multifaceted position towards Deaf community. On the one hand, we are privileged in terms of access to knowledge, our level of education and our position within the academic world. On the other hand, we consider ourselves outsiders in the Deaf community. While we share cultural awareness and participate in various local Deaf events, we lack the lived experience of deafness.

Because of organizational and budget constraints, we were not able to ensure presence of Deaf people in the research team throughout the entire project. However, we implemented elements of participatory research into our study (Anderson et al., 2018; Smits et al., 2020). We consulted Deaf people and sign language interpreters during the design of the survey, as well as the pilot study and its analysis. We also discussed the dissemination of the survey with them. Then, we invited six Deaf individuals with diverse backgrounds to form an advisory team. RB, BB, JK and KR are Deaf educators and activists working at local non-governmental organisations and universities. JK and KR are also PJM interpreters. BK and FK are members of local Deaf community. We collaborated during the data analysis and manuscript preparation (see the Methods section), aiming to discuss the ways in which our—hearing and Deaf—perspectives interact with the responses of Deaf people, and to critically consider our assumptions during these processes. Moreover, we aim to extend the joint work during dissemination of the results.

## Methods

This paper presents qualitative analysis of the data generated through an online survey. A protocol of this study was preregistered and shared at the Open Science Framework (OSF: https://osf.io/vfsu7). We present the outcomes with regards to the Standards for Reporting Qualitative Research (SRQR; O’Brien et al., 2014, Supplementary file 1). We were interested in exploring contextually situated perspectives and experiences of our respondents. We acknowledged the active role of researchers within the qualitative analysis and its creative nature—the role of our values, assumptions, culture, as well as our bodies in shaping our experiences and interpretations of the data (Braun & Clarke, 2025; Hall, 1997). We also acknowledged the need to stay close to experiences of our respondents—Deaf people. Thus, we took the position of transformative paradigm with elements of participatory research, providing space within our study to express marginalized perspectives (Harris et al., 2009; Mertens, 2017; Smits et al., 2020).

### Sample and recruitment

Deaf people in Poland are often excluded from the participation in social life, facing lack of access and showing distrust towards initiatives of hearing people (Marganiec, 2024). Moreover, they are scattered over the whole country, with larger communities present in main cities. Thus, we followed a purposeful sampling approach (mixed *criterion/snowball sampling*), which employed minimal inclusion criteria to the study (Patton, 2002). We invited Deaf people aged above 18 years, who had previous experiences as research participants, and/or had interests in research participation, and/or were Deaf community activists. We considered deafness from the socio-cultural perspective, paying the attention to the command of sign language and identification with Deaf culture (Cue et al., 2019; Friedner & Kusters, 2020). The key criterion was self-identification.

To reach a broad sample of Deaf people we used various recruitment methods: we contacted several NGOs revolved around the life of Deaf people, one *Deaf studies* university department, as well as published the survey on the online groups for Deaf people and did snowballing using personal networks. We planned to reach about 50 respondents. We assumed that such amount of data will have sufficient *information power* to allow for in-depth analysis (Malterud et al., 2016; Terry & Braun, 2017), but were open for later adjustments. As a form of incentive, respondents received vouchers valued 200 PLN (about 50 USD). We sent the vouchers to respondents via email after receiving the notification about completing the survey.

### Ethics

The study was approved by the Jagiellonian University Medical College’s Research Ethics Committee (No: 118.0043.1.60.2024). All respondents provided informed consent to participate and process their data through an online consent form. They were assured that they could withdraw from the study at any point or withdraw their data afterwards. Respondents were also encouraged to contact the authors in case of concerns they might have about the study. The personal data were processed with regards to the General Data Protection Regulation (GDPR). The informed consent and GDPR information were bilingual—in written Polish and PJM.

### Data collection

We conducted an online open-ended survey (Knutsen & Presser, 2010). It enabled us to both reach a broad sample of Deaf people from diverse regions and adjust the mode of answering for providing deeper responses (Anderson et al., 2018; Terry & Braun, 2017). The survey consisted of two parts (see: Supplementary file 2). The first part included eight open-ended questions about research experiences of Deaf people, their preferences, needs and opinions about research accessibility. The second part included eight questions about demographic and socio-cultural characteristics of the respondents (for detailed overview see Table 1). We used the MS Forms online platform. The survey was piloted on a sample of Deaf people who have previous research experiences (n = 12). After discussions with respondents, preliminary analysis and minor adaptations of the demographic part, we disseminated the survey. As there were no changes to the open-ended questions we included the pilot in the final analysis.

**Table 1:** Characteristics of the respondents (N = 52).

Characteristics	N	%
**Gender**		
male	21	40%
female	28	54%
non-binary	1	2%
prefer not to disclose	2	4%
**Age (range)**		
18-39	39	75%
40-59	13	25%
60+	0	0%
**Education**		
primary	1	2%
secondary	24	46%
high	23	44%
other	4	8%
**Socio-cultural identity**		
Deaf	31	60%
deaf	3	6%
hard-of-hearing	6	12%
hearing	2	4%
bicultural	7	13%
other	3	6%
**Preferred form of communication**		
PJM	34	65%
SJM^*^	2	4%
Polish (spoken)	5	10%
other	11	21%
**Written language competency (Polish)**		
very good	23	44%
good	21	40%
moderate	6	12%
poor	2	4%
very poor	0	0%
**Sign language competency (PJM)**		
very good	36	69%
good	10	19%
moderate	4	8%
poor	1	2%
very poor	1	2%
**Participation in research**	*n = 40*	*n = 40*
never	3	8%
once	6	15%
few times (2-10)	24	60%
many times (> 10)	7	18%

^*^SJM (*System Językowo-Migowy*) is a signed system representing grammar of spoken Polish, not being a language itself.

When designing the survey, we attempted to balance the factors which could impact the willingness of Deaf people to participate in our study, led by hearing researchers (Anderson et al., 2018). The survey was bilingual. The welcome information was signed by TK to introduce the hearing team to a broader Deaf audience, as well as explain the study aims and reasons behind it. Respondents could choose between the text-only Polish version or the bilingual Polish-PJM one, with questions translated by a Deaf sign language interpreter—RB. They could also respond to the open-ended questions bilingually, either with writing answers or uploading a signed recording for each question. It gave the respondents open choice between the greater anonymity (written language) or linguistic preferences (sign language).

Overall, between July and October 53 people filled the survey (12 from the pilot study and 41 from the final version). After preliminary analysis of the results, we decided that these data had sufficient information power, and no further responses were required. We excluded one person because of double-filling the survey. Eventually we included 52 answers. Data analysis had two steps. In the first step we prepared an extraction sheet for socio-demographic data, presented in the Table 1. In the second step we analysed the open-ended answers using the reflexive approach to thematic analysis (Braun et al., 2019; Braun & Clarke, 2021). We conducted whole qualitative analysis in MaxQDA24 software (version MaxQDA24.7.0). We analysed the data bilingually, based on written transcripts and/or sign language videos.

### Data generation

We followed the experiential orientation to reflexive thematic analysis (Braun & Clarke, 2021). It allowed us to develop both semantic and latent patterns of meaning grounded within experiences of the respondents. We employed a primarily inductive approach to coding. It was influenced by the Deaf studies, disability studies and participatory research literature, as well as our research experiences and contacts with the local Deaf community. However, we did not attempt to fit the data into these frames (Braun & Clarke, 2021, 2025).

Whole process—familiarisation, coding, developing, reviewing and refining themes, writing the report—was conducted by TK (Braun et al., 2019; Braun & Clarke, 2021). Each stage was regularly discussed with hearing co-researchers, and—since the coding phase—with the Deaf advisory team. These discussions aimed to enrich the process and centre the perspectives and lived experiences of our respondents (Braun & Clarke, 2021, 2025). While it was not feasible to seek feedback from the respondents, we will disseminate to them bilingual summaries of the results. Throughout the process TK was collecting field notes concerning his experiences, feelings and reflections about the research. During familiarisation we noticed potential points of interest and connections to the literature (e.g., Anderson et al., 2023; Krawczyk et al., 2024; Singleton et al., 2014). Originally, TK coded hundreds of fine-grained codes which we later grouped into a more manageable amount. We grouped codes into 13 preliminary themes. We discussed them with the Deaf advisory team and refined them into seven themes. BK and FK proposed the final theme structure, which reflects the path from the current state of things, the needs and goals of our respondents towards the ways to overcome them. Finally, we reframed the seventh theme “Communication” into an overarching theme, as its various aspects were present through all the data. Also, we changed its name to “Dialogue matters” to emphasize relational aspects.

We conducted the analysis in Polish and PJM. We translated all chosen quotes to English while preparing the manuscript. Written language is often a second language for Deaf people, and their writing style can reflect the grammar of sign languages (Anderson et al., 2018; Tomaszewski & Moroń, 2018b). During translation we followed the general grammar style of our respondents, but did not recreate spelling errors. We also stayed true to the form of (non-)capitalized word “deaf”/“Deaf” used by the respondents.

## Analysis

Our coding tree consists of six themes under one overarching theme. We present them as a pathway from the present towards the desired state, also outlining the necessary conditions and potential means to achieve them: (i) *We want to develop research among Deaf people*, (ii) *Science is not accessible to us*, (iii) *We have had bad experiences*, (iv) *Science gives new opportunities and perspectives*, (v) *We want full accessibility*, and (vi) *Let us establish trust*. The themes highlight the issues of social and epistemic (in)justice for Deaf people, as well as the need for greater research accessibility. The issue of communication recurred frequently across various themes. It concerned not only accessibility of communication but also open and respectful relations with Deaf community, individual participants, and co-researchers. Thus, we brought together all themes under one overarching theme: Dialogue matters.

### We want to develop research among Deaf people

Our respondents expressed the need and desire for greater participation of Deaf people in scientific enterprise. They were aware of the research’s role in contemporary life and interested in scientific topics. They also recognized the need to popularize interests in science among Deaf people and disseminate research results in an accessible manner:

I just want to say that this research [on accessibility] is very interesting and very much needed in our environment to better understand the specific needs of d/Deaf people in the research area​​. Few people are interested in the accessibility of surveys or in general research for d/Deaf people – so thank you for having it here! :-) (P6)

Moreover, our respondents highlighted the importance of increasing research engagement within the Deaf community: “Every Deaf person should participate in research to express their voice, their experience and their opinion on a given topic” (O48). This issue arises because, within the Polish Deaf community, a small number of individuals are actively engaged in numerous initiatives, including research. At the same time the rest of the community remains barely or not engaged at all. However, in recent years civil activity of Deaf people has increased (cf. Marganiec, 2024).

Among research areas considered as important were the accessibility, education, physical and mental health of Deaf people, as well as sign languages, Deaf culture, and relations in Deaf communities. Our respondents rated the importance of specific topics differently, and also varied in regard to the awareness of the already existing research. Nonetheless, they agreed that there is a need for more research for the Deaf community to flourish. As one respondent stated: “I think that every topic is needed. We have so little research among the Deaf” (P1). To achieve this goal, the respondents emphasized the importance of collaboration between Deaf and hearing people in research. Such collaboration should happen on equal terms, where Deaf people could also serve as researchers, consultants, experts, and project leaders:

It is good to cooperate with the d/Deaf, select Deaf people to some position occupying a research specialist, thanks to which they have greater experience and communicative language, knowledge of PJM, awareness of the d/Deaf environment. (P5)

Additionally, our respondents believed that Deaf people would be more eager to participate in research and would have greater trust in scientific institutions if research were conducted by Deaf scientists. Thus, more Deaf researchers are needed:

If research is aimed at the deaf community, I would feel good when the researcher listens to a deaf person or cooperates with a deaf person, thanks to which they understand the deaf community well and can actually conduct research. (O39)

### Science is not accessible to us

While recognising the importance of and need for research, our respondents shared the feeling that Deaf people are excluded from scientific activities. They perceived academia as a “hearing space” and discussed the inaccessibility of research, scientific information, and scientific events:

What discourages me the most from participating in research is the lack of adaptations for Deaf people. If there is no PJM interpreter or sign language materials, it is difficult for me to fully understand what’s going on in research. The lack of clear communication makes me feel excluded. I also give up when the research is complicated or unclear and the researchers don’t understand Deaf people’s needs. Even if the topic is interesting, the lack of support and access to translations makes participation difficult and stressful. (O38)

One of the most urgent issues among these barriers was the lack of information. It relates both to “the lack of adequate information networks” (O23) regarding research opportunities and the insufficient information about the conduct of specific research. Followingly, our respondents frequently emphasized communication barriers. Written/spoken language is often a foreign language for Deaf people and scientists use “…some terminology, which may be incomprehensible even in the simplest way of explaining in PJM. Often it may be that we [think that we] understand but then it turns out that it wasn't quite the point” (O29). The use of scientific jargon can make scientific texts difficult to understand, even when translated into the sign language (e.g., because of the lack of scientific vocabulary in PJM, the difficulties with explaining specific terms or regional differences in signs).

Furthermore, Deaf participants can encounter “research materials, such as surveys or questionnaires, often not adapted to Deaf people’s needs, which may affect the accuracy and reliability of the data obtained.” (O14). This issue relates to both quantitative and qualitative methods (Talipska, 2024). It can also reduce heterogeneity of the results, because “Deaf people who don’t know Polish well aren’t able to express themselves, but [they] may have more interesting or different experiences on a given topic” (O48). This leads to the exclusion of the perspective and experiences of Deaf sign language users who do not have good competences in written language and over-researching of the well-educated, bilingual members of the community.

Depicted factors also relate to the fact that most scientists are hearing, even in the field of Deaf studies. Our respondents suspected that if scientists had little or no contact with Deaf communities, they would lack cultural awareness and knowledge about Deaf people and their life. Thus, such scientists could not know how to enable Deaf people’s full participation in research. Our respondents also reported unsatisfactory cooperation with research staff and lack of their efforts to ensure accessibility. As one respondent stated, presented situation can also cause methodological errors, poor quality or inadequate interpretation of research:

If the research is focused on the d/Deaf Community and the person who is doing the research is unknown to anyone, then I have a negative attitude – such research makes me think of a guinea pig. Because this person doesn’t live with the Deaf Community on a daily basis and then I have some doubts whether the research is reliable, conducted correctly, whether [researcher] understands our Deaf Culture, etc. (P1)

These factors make Deaf people feel excluded and overlooked in research and science, which lead to variety of negative emotions, presented in the next theme.

### We have had bad experiences

The presented situation evoked a diverse range of negative feelings and memories among our respondents related to their past research participation and interactions with researchers. As one respondent stated, “It's hard to answer [the question about the perceived difficulties in research] because everything was difficult for me” (P9). Research participation was frequently associated with the feeling of uncertainty and discomfort, stemming from the research inaccessibility and the lack of cultural awareness among hearing researchers:

What discourages me the most from participating in scientific research are situations that cause discomfort, lack of understanding […] If I am not sure that the information will be conveyed correctly, I feel uncertain, which discourages me effectively. If researchers aren’t empathetic or seem unprepared to work with d/Deaf people, I lose the will to cooperate. (O14)

Taking part in research also entailed fatigue and psychological efforts. For instance, social sciences research can require reexperiencing difficult memories:

I don't see any point in sharing something difficult (my personal difficulties, my experiences with communication barriers, or rejection by the Hearing Community) with someone who doesn't know my world. (P1)

Both kinds of experiences evoked resistance not only to continued participation in the specific study, but also to the involvement in future research. In other cases, complex research procedures carried out in unpleasant and incomprehensible settings by unknown, non-empathetic hearing researchers made our respondents feel observed, judged, or even dehumanized:

Being in a “study” makes me feel like a guinea pig, being observed from all sides, I am a human being just like you, but I feel like I am being put under a microscope and examined from all sides. (O25)

Such feelings interplay with a general distrust of hearing people and their actions, as well as with the ongoing exclusion that Deaf people experience in everyday life, in the face of the hearing dominance. In research settings our respondents faced audism—discrimination due to their lack of hearing (Gertz & Boudreault, 2016). They encountered hearing researchers who manifested a sense of superiority or had unreasonably low expectations of Deaf people. Audism has also a structural form, which creates systemic advantages for hearing people and reinforces the prejudice against Deaf people (Gertz & Boudreault, 2016). Our respondents experienced this kind of audism in research and at scientific events too. The medical model of deafness dominates in social awareness, also within academia, which influences, for example, the range of topics considered as “worthy to research” and “serious issues.” Moreover, one female researcher mentioned the existence of glass ceiling for Deaf scholars in academia:

Research topics related to the deaf are not always accepted, treated more as curiosity than a real research problem. […] very often the voice of hearing researchers dealing with the deaf, who don’t know sign language, who don’t know the deaf culture, is heard and accepted. (O13)

Participation in research was associated with various organizational efforts, such as arranging lengthy and complicated transportation to the research site or ensuring childcare. Our respondents frequently emphasized the time-consuming of research activities which discouraged them from participation:

Honestly, many people don't like filling out a survey because they think it's a waste of time. That's why it's better to have a specific topic, questions marked e.g., “yes”, “no” or “I don't know” to mark. So that one can answer relatively quickly and that it doesn't drag on for up to 1 hour in filling out because who can bear? (O34)

Our respondents also felt discouraged by recurring research topics and lacking information about research results or intended outcomes: “When I see that research has no specific goal for the future and is done 'for fun' or 'for curiosity', I don't complete such. […Such research] makes me feel like I'm wasting my time” (O20). Despite existing barriers and required efforts, there were researchers who did not inform participants about research objectives or its potential benefits. All these factors contributed to the reluctance of many respondents to participate in research and to their lack of interest in it. This led them to perceive research as yet another burden: “If there is a negative feeling, I don’t want to continue, what for... Each day I have various barriers and problems, so for more I quickly “thank you” and also it takes up my valuable time” (O36).

### Science gives new opportunities and perspectives

Despite the negative experiences, our respondents perceive science and research as a valuable source of benefits for both Deaf and hearing people. Participation in research and academic life allowed for broadening knowledge by learning about new discoveries and enabled access to new technologies. Research can be a platform for exchanging information, both among Deaf people, and between Deaf and hearing people. Also, it “will allow us to get closer to such actions and choices in life that will give us the greatest chance of success” (O43). Thus, science contributes to mutual development of Deaf and hearing people. On the one hand, research participation is beneficial for individual participants, allowing them to “gain new information and better understand the world around me, including what is happening in the social, health, economic and other areas” (O30). On the other hand, research contributes to the social change of the whole society and improvement of coexistence between Deaf and hearing:

I am often motivated by an inner desire to contribute to science, to help others, or to feel that my voice matters. Knowing that my research participation may benefit the Deaf community, or the wider community is a strong motivator for me. (O14)

Altruistic factors—the “willingness to help and solidarity” (P6) played an important role in decisions about research participation. Especially the possibility to support and empower the Deaf community, specific Deaf researchers, or initiatives led by Deaf people was an important motivator:

Changes, if research can bring some change in the deaf world, then I always take part in them. I experience various difficulties in everyday life, I know how it is, changes are needed, therefore even if the research is to help other people see how we live, then maybe it will help us to solve a few problems. (O25)

Research participation allowed our respondents to express their perspectives and experiences, which can help to raise social awareness of Deaf community, as well as combat discrimination—science “could change a lot, and their [hearing] approach to deaf people – treating them as adults, not treating us like a mentally disabled person or a small child” (O21). Moreover, through research participation Deaf people can enhance their self-efficacy and empowerment: “I feel that I can change something thanks to this [research], I express my views, I show how [it] really looks like from the perspective of a deaf person” (O16). Participation can thereby positively affect individuals’ well-being by providing satisfaction and fulfilment through contributing to the advancement of science or improving social life:

What encourages me the most, is that I see that some can contribute to a great change in our society. And to the expansion of our knowledge, which can be very valuable. Gaining such great experiences is something sacred to me. (O17)

### We want full accessibility

Our respondents emphasized the need for greater research accessibility and the struggle for full participation. The main issue mentioned was the use of sign language which “gives the possibility of full understanding [of] the research purpose. More people can take part in such research and feel appreciated, not omitted in this important issue” (O40). Linguistic accessibility can be ensured either by providing the translation or by establishing sign language as the working language of research (cf. Anderson et al., 2018). The quality of signing, which depends on the qualifications and working conditions of interpreters (or researchers), is important. Further, our respondents stressed the need for using plain and understandable language, which allows for comprehension of the research aims, procedures and content. Especially, they expected “that the purpose of the study is clearly defined and transparent” (O14). These issues were considered as main factors contributing to full participation. Some respondents preferred bimodal information, while others just using the sign language:

To explain, write… For some Deaf people subtitles are convenient, then they understand more, and for others subtitles aren’t helpful, they feel comfortable when signing, it depends on the person. […] I prefer signing. Subtitles are convenient for some, but I don't know what is going on in subtitles. If someone explains it to me, I know and I can answer quickly. (O26)

Therefore, it is important to provide participants “the possibility to choose the form of questions and answers” (O52). This reflects one aspect of the diversity present within Deaf communities—heterogeneous linguistic preferences (Wilson & Winiarczyk, 2014). Our respondents frequently emphasized the need to adapt research to specific needs of participants. Thus, reaching participants before the research to discuss their needs plays an important role. Also, respondents pointed out that the entire research process—from planning and designing to results dissemination should be accessible and/or open for active participation:

[Deaf people] should not only be research participants, but also take an active part in its design, analysis of results, and implementation of conclusions. This will enable research to be better tailored to their real needs. (O33)

Therefore, our respondents suggested planning additional resources and time to ensure research accessibility. Depending on the study character, researchers should provide additional support and materials, aligned with the spatial–visual cognition mode of Deaf people, such as the “use of visual materials such as graphics and illustrations, films” (O32), brochures, “some kind of glossary - what difficult, specialist words mean” (P7), also, “there can be several examples, 1-2 is enough, practical ones” (P4). Accessibility would also be enhanced by short duration of the study and providing compensation, so that participants can balance participation with personal or family responsibilities.

### Let us establish trust

Finally, our respondents shared the sense of mutual trust as the condition for full participation of Deaf people in scientific activities. The relationship between the participant and researchers or between Deaf and hearing research team members plays a key role: “Everyone should feel good, comfortable, so it is important that the person leads the conversation in a pleasant atmosphere. […] Understanding and finding a common language” (P7). Research team should be perceived as ethical and trustworthy, aware of the Deaf community norms and rules, as well as familiar with and respectful of sign language and Deaf culture:

Empathetic, friendly and well-trained researchers have positive influence on my decision to participate. […] it is important that the interpreter is perceived as a trusted person who cares about the confidentiality of the information provided. This will allow participants to express their opinions and experiences openly and honestly. (O14)

Moreover, researchers’ proficiency in sign language gave the respondents comfort and confidence that they would be understood properly: “I think that the greatest comfort is comprehensible communication, so the presence of PJM - i.e. a PJM interpreter, a researcher who knows PJM fluently” (O29). This allows participants to be open and build relationships with researchers. Our respondents emphasized that the contact with Deaf participants should not be limited only to the duration of research (this reflects general guidance on research involvement & engagement, cf. Vincent et al., 2022; World Health Organization, 2020). Prior presence of researchers in the community and regular participation in its events are important for building a relationship:

I believe that research concerning the d/Deaf must be based on a long, close relationship between the researcher and participant. […] if the research is conducted by the d/Deaf or hearing researchers who know PJM and know the Deaf Society, then I have positive attitude, it connotates sincere, honest research. (P1)

Perception of the researcher as a community insider positively influences Deaf people’s willingness to participate in research, especially if the researcher is Deaf. This may require considerable efforts from hearing scientists, both in terms of time and additional resources.

Within research relationships, our respondents emphasized the role of privacy and anonymity, as “the Deaf world despite the introduction of reliance clause [GDPR] is not so anonymous” (P12). The small size and closeness of Deaf communities, alongside the visual character of sign language pose significant risks—“if a deaf person has to record a video, it is automatically not private” (O45). Many Deaf people are anxious about their and their relatives’ privacy. Thus, a trusted research team and appropriate data protection—especially for data recorded in sign language—are crucial for the honesty and openness of participants. Our respondents proposed several solutions for reducing these risks, for example:

To organize a person who will act as a mirror for Deaf individuals responding to surveys. This means a Deaf person signs, and another person mirrors what the Deaf person signs, including signing directly to the camera. This setup requires connecting online or in person. (O45)

Ensuring such or similar precautions can contribute to minimising the privacy risks. All these factors contribute to the importance of building trust, preferably before the research begins.

### Dialogue matters

We raised the issue of communication as an overarching theme due to its frequent recurrence—both explicitly and implicitly—in a variety of contexts, both negative and positive ones. When sharing negative memories about the contact with hearing researchers and experienced discrimination, our respondents emphasized the lack of successful communication and willingness to dialogue on the side of hearing researchers. Furthermore, recollected barriers towards full research participation often concerned inaccessible communication and linguistic issues. Thus, in research they were both deprived of the means for successful communication and encountered reluctance or unawareness towards engaging in its respectful process.

Also, good communication and relations were key factors behind a successful collaboration. When basic communicational means are provided and researchers are willing to collaborate, this contributes to the interest in research and increases the willingness to engage. Moreover, respectful communication is a basis for establishing relations between hearing researchers and the Deaf community. It is crucial for mitigating distrust towards hearing people, often present in Deaf communities. Also, our respondents emphasized the role of two-way communication during the entire research process, beginning with the provision of sufficient research data before the study, then informing fully about research progress, and finally, disseminating research results to the community. Thus, communication and dialogue are a basis for research cooperation, a factor which mediates the process, and a result of research activities.

## Discussion

Our study provides insights to Deaf people’s perspectives on and experiences from research participation. It is one of the few which presents the data from outside the Western, English-speaking countries (Krawczyk et al., 2024). Being Deaf in research was presented with both the negative and positive sides. Our Deaf co-researchers’ suggestion to present experiences of our respondents as a pathway, mirrors Deaf scholars’ views on *being d/Deaf* in general as an *odyssey, journey,* or *emancipatory path* (Cue et al., 2019; Podgórska-Jachnik, 2013). The path of our participants starts in history, with memories of facing audism in research, as well as the lack of research accessibility and cultural awareness of (hearing) researchers. Then, we may see the current state as transition time (Tomaszewski & Moroń, 2018a). On the one hand, stereotypes and prejudices towards Deaf people still persist, alongside inaccessible research processes and procedures. However, the number of culturally aware hearing researchers increases, along with the number of Deaf (co-)researchers and scholars. There is an interest in research among Deaf people, with increasing awareness that research on deafness is a *Deaf thing*. It gives a hope, that research and academia will become more diverse and open for various minority groups, such as Deaf people.

The presented situation reflects, on the one hand, the general life experiences of Deaf people around the world—being torn between struggling with hearing-oriented reality and embracing Deaf identities (Cue et al., 2019). On the other hand, it mirrors Polish perspectives on the present as a time of transition and redefinition of Deaf identity, shaped both by the recognition of sign languages as natural languages, and by advances in hearing aid technology and screening methods (Tomaszewski & Moroń, 2018a, 2018b). This context prompts a reconsideration of the American model of Deaf culture—adopted by the Polish Deaf community over the past two decades—within the local conditions (Tomaszewski & Krzysztofiak, 2025; Zdrodowska, 2019).

Our respondents subscribe to the common view of research and academia as spaces dominated by the hearing-oriented expectations and sociocultural norms, described by Listman & Dingus-Eason (2018, p. 280) as the *phonocentric hegemony*. But, the scale and frequency of issues discussed may reflect the local context. The most frequent issue concerned the basic needs, such as communication access, need for bilingual research materials and the recognition of sign language in research. Subsequently, our respondents emphasized the role of the contact between Deaf and hearing people, establishing relations and trust as a base for meaningful scientific collaborations (cf. Anderson et al., 2023). Then, they extended the discussion further to the issues of sociocultural awareness and competency between Deaf and hearing people. Such gradation reflects a persistent general lack of awareness of Deaf culture at most Polish universities, with a few notable exceptions (Talipska, 2019). This situation necessitates a continual struggle for basic accessibility measures in many contexts. At the other end of the spectrum are a few accessible research projects conducted by socio-culturally oriented researchers, for example, from the Section for Sign Linguistics at the University of Warsaw—the only institutional body dedicated specifically to sign language and Deaf culture research.

Accessible research conducted by linguistically and culturally competent researchers allows Deaf people to cease *changing themselves* in order to “fit into” research and enable them to *be themselves* and express themselves fully (Talipska, 2024; Young et al., 2000, pp. 189–192). This includes not only inclusive research processes and adapted research materials, but also reconsideration of existing academic spaces and procedures (Ferndale, 2018; Listman & Dingus-Eason, 2018; O’Brien, 2020). Accessibility is a baseline from which all meaningful collaborations can begin and develop, both between research team members, and between researchers and participants. Thus, it is a pillar of scientific partnership on equal terms, alongside the recognition of Deaf epistemology as a valid form of knowledge, and the recognition of Deaf people as knowledge owners (Harris et al., 2009; Holcomb, 2010).

Increasing awareness and knowledge about sociocultural aspects of deafness is also crucial, as hearing culture—consciously or not—follows the medical, disability perspective on deafness (Cue, 2024). Skyer & Cochell (2020, p. 9) argue that it may result not only in erroneous epistemological views of deafness, for example, Deaf people having limited capacities to learn (thus, to participate in or conduct research), but also in the ontological flaw—perceiving *being deaf* as broken, in need of correction and support. Such views may disturb establishing meaningful collaborations between Deaf and hearing people even stronger than research inaccessibility, impeding the possibility of dialogue and mutual understanding. Recognition of Deaf epistemology relates to the calls for active participation of Deaf people in scientific enterprise (Tomaszewski & Krzysztofiak, 2025). It also requires hearing people to recognize their limits regarding their understanding of deafness (Benedict & Sass-Lehrer, 2007), and academia to “give deaf scholars the space and opportunities to reclaim deaf knowledge and show deaf expertise” (Henner et al., 2021, p. 237). It would not only mitigate the risk of perpetuating harms towards Deaf community but also allow for gathering more adequate and richer knowledge (Wolsey et al., 2017; Young & Ackerman, 2001).

Our respondents acknowledged efforts related to creating conditions for meaningful collaboration. Accommodating Deaf people *in* research and *as* researchers in an ethical manner—thus allowing them to *be themselves—*entails that hearing researchers and research institutions need to *change themselves* (Young et al., 2000). These changes involve additional planning and resources, including efforts to secure more time and funding throughout the research process, as well as the adaptation of academic procedures, research dissemination, and infrastructure (Ferndale, 2018). Epistemic efforts are equally important, including the development of personal and institutional awareness, knowledge and competences (Krawczyk et al., 2025). Hearing researchers should also recognize the impact of a difficult past and harms done in research to Deaf communities (Anderson et al., 2023). While effortful, such practices may not only empower the engaged group, but contribute to more accurate and applicable knowledge (Harting et al., 2022; Krawczyk et al., 2025).

We want to emphasize the diversity around and within the Deaf community. Our respondents mentioned the need for including other marginalized perspectives, among them those of CODAs (hearing Children Of Deaf Adults), hard of hearing, and Deaf-Blind people (cf. Wright, 2021). Each group and each person within these groups may have different needs that differ to various extent from those of Deaf people. Also, each Deaf community may have its own level of Deaf culture awareness or view of an “ideal” Deaf individual (Pudans-Smith et al., 2019; Tomaszewski & Krzysztofiak, 2025), as well as different experiences and needs (Jones et al., 2021; Wilson & Winiarczyk, 2014). On the one hand, our respondents expressed a similar scope of issues to the participants from Western countries (sign language access, cultural competences, negative experiences, see e.g., Anderson et al., 2020; Singleton et al., 2014). This may relate both to universal experience of facing similar barriers, and to the adoption of the American model of Deaf culture in Poland (Zdrodowska, 2019). On the other hand, mentioned issues differ in their frequency (e.g., dominance of accessibility-related issues) and specific topics (e.g., infrequent genetics research topics), which may relate to the interests of specific respondents and regional differences (e.g., modest debate about genetics research at the national level).

Intragroup diversity relates also to the range of needs—for example the preference of sign language versus bilingual materials. Our respondents praised the survey for the possibility of choosing the language. However, we were surprised by the low usage of the bilingual form (46%). Moreover, only 3 respondents (6%) decided to provide recorded signed answers instead of written ones. It contrasts with the frequent calls for bilingual or sign language only research. Such calls were expressed also by the respondents using written-only option. This issue may reflect the well-educated profile of our respondents (secondary education: 46%; higher education: 44%) or frequent critique of time-intensive studies. Our respondents were in majority big “D” culturally Deaf individuals (60%). While they preferred the use of sign language (65%), the majority rated both their sign and written language competencies as very good and good (respectively, 69% and 19% for PJM, and 44% and 40% for written Polish). Watching signed recordings requires more time than reading if the reader is familiar with the written language. However, it doesn’t explain fully why over the half of respondents didn’t use the bilingual option, even just to comment and criticize it.

### Limitations

The study had several limitations. First, the online form can be considered both as a strength and a limitation. It allowed for reaching a broad sample from various regions of Poland. However, it may have posed a barrier for older Deaf people who are not familiar with modern technologies (75% of our respondents were aged 18–39), as well as for Deaf people with limited skills in any formal language (e.g., users of home sign). Furthermore, it may have reduced the depth of the answers, as there was limited possibility to establish trust between the participant and researchers. Our hearing-led research team might not have encouraged respondents to provide video recordings in PJM showing their clearly identifiable image, which so far cannot be de-identified for the analysis. Thus, we may have excluded Deaf people with lower level of written language competence who were unwilling to share their image. This, however, still remains an issue if in-situ research is recorded for analysis. These factors may explain the low number of signed responses. Finally, within the cooperation with Deaf advisory team we faced organizational constraints. Most Deaf members have full time jobs and some of them live in remote locations. It limited the ability to conduct jointly the full scope of thematic analysis. But, we discussed together the most important steps which enriched the analysis and provided us knowledge about nuances concerning, among others embodied experience of deafness and negative reception of some superficially neutral wordings.

### Implications

While we are cautious about favouring some communities over another when comparing various countries and regions, we argue for facilitating discussions and transfer of knowledge on how to create inclusive society and research. Deaf people over the world are united by experiences of marginalisation and life within the *phonocentric hegemony* (Listman & Dingus-Eason, 2018). Thus, we argue for collaboration and exchange of good practices between research and academic accessibility centres, not only at regional and national, but also the international level.

Also, we argue for more locally contextualized co-designed projects about the research accessibility (cf. Greenhalgh et al., 2019). While from the global perspective such projects may not provide ground-breaking results—outlining likely similar patterns of hearing dominance and Deaf struggle, they can highlight important local differences and nuances. Moreover, they may be a crucial step towards full participation in research and empowerment of local Deaf communities, as well as reclaiming the ownership over their knowledge and epistemology (Cue et al., 2019; Greenhalgh et al., 2019; Holcomb, 2010).

We want to emphasize again the diversity within and around the Deaf community, and the need for including other marginalized perspectives. Thus, while general guidelines about collaborative research with Deaf people may be useful as a basis, it is crucial to reach the specific group before the study (preferably during study design) to discuss their specific needs. This study will be a basis for creating such recommendations, situated in the Polish context. We plan to provide a form of executive summary which will be available for researchers and Deaf community members. Also, we argue for research conducted according to the co-creation or participatory approaches, whenever such conduct is ethically feasible and reasonable (Greenhalgh et al., 2019; Singleton et al., 2017).

## Conclusion

Deaf studies remain dominated by perspectives of hearing people, despite broad discussion in the field. While we contribute to that picture, we also wanted to provide space for Deaf people to express their perspectives. Our respondents discussed several issues, from research accessibility to sociocultural awareness of deafness and Deaf culture. They emphasized the role of communication and willingness to dialogue. While these factors may seem general and vague, each of them can be divided into small, precise actions. We hope that our study will inspire such practical, collaborative actions aiming towards ethical research inclusion of Deaf people. This also requires academia to critically consider its attitudes towards various lay partners, such as Deaf people, to be able to establish fruitful collaborations on equal grounds. We hope that such enterprise is possible when both sides are open to cooperation and reflection on owned biases and preconceptions. As ethical and accessible research is also about dialogue.

## Supplementary Material

SRQR_Checklist_enag006

Summaries_text_for_translation_enag006

Survey-questions_enag006

## Data Availability

The survey data underlying this article cannot be shared publicly due to the privacy of individuals that participated in the study. The data will be shared on reasonable request to the corresponding author. All data that can be disclosed under the GDPR are available in the article and in its online supplementary material, as well as openly available in Open Science Framework at https://osf.io/9akm5/.
