# Supplementary material for: Dialogue matters. Exploring Deaf people’s research experiences in Poland
Source: J Deaf Stud Deaf Educ. 2026 Feb 23;31(3):460–70. doi: 10.1093/jdsade/enag006 (PMC13343195; doi:10.1093/jdsade/enag006)
Supplement: Summaries_text_for_translation_enag006 [file summaries_text_for_translation_enag006.docx]

**Dialogue matters. Exploring Deaf people’s research experiences in Poland**

Despite its dynamic development, science is still often inaccessible to minority groups, such as Deaf people who use sign language. The Deaf community is diverse in terms of identity, language preferences, life histories, and experienced discrimination. Nevertheless, researchers are rarely interested in the experiences of Deaf people regarding their participation in research. Therefore, we wanted to explore the experiences, opinions, and needs of Polish Deaf people concerning their research participation. We also aimed to include the perspectives of Deaf people from Poland in the academic discourse.

We conducted a qualitative study – an online survey with open-ended questions. The survey was bilingual - in Polish and Polish Sign Language (PJM). We received 52 responses from various parts of Poland. Most of the participants was PJM users and had a good command of both PJM and written Polish. Since all members of our research team are hearing, we invited a group of Deaf individuals to collaborate during the analysis of the results. We wanted to jointly discuss how we understand and interpret the experiences of participants. We used the thematic analysis, following the Braun and Clarke’s approach. It enabled us to present an in-depth picture of the experiences of Deaf people. Together, we developed six themes, arranged as a path, from the history, through present times, toward the future.

The findings reveal an interest among the Deaf participants in research on topics related to deafness and the Deaf community. They describe the current situation – the inaccessibility of science – including not adapted procedures, a lack of cultural awareness among researchers, and experiences of audism – discrimination by hearing scientists. This situation evoked negative emotions, discomfort, and distrust toward hearing researchers, making research participation psychologically demanding. Despite these negative experiences, participants perceive science as a source of new opportunities. Research contributes to the development of Deaf and hearing people, strengthening the Deaf community, combating discrimination, and raising social awareness. It gives Deaf participants a sense of self-worth and satisfaction from contributing to the scientific progress and improvement of social life.

Participants emphasized the need to ensure accessibility in science – bilingual materials adapted to the needs of diverse Deaf participants. Accessibility is also supported by clear explanation of the purpose and intended outcomes of research, as well as by offering compensation. Research collaboration should be based on trust. Researchers should be present in the Deaf community and aware of its norms and customs. This allows for relationship building and openness during the research process. In the small Deaf community, confidentiality and anonymity are also crucial. Participants repeatedly spoke about accessible and respectful communication, which is why we concluded our analysis with the overarching theme: Dialogue matters.

We hope that our study will contribute to the improvement of research accessibility and ethics, as well as inspire research collaborations between Deaf and hearing. The pillars of such cooperation are:

- Accessibility of the entire research process,
- Cultural awareness of researchers, and
- Active participation of Deaf people in science.

Such collaboration should be based on dialogue, mutual respect, and understanding. This requires the scientific community to critically examine its attitudes toward various social groups, such as Deaf people. We believe such a process is possible if both sides will be open to cooperation and reflection on their own assumptions. We recommend supporting research projects involving local Deaf communities and international exchange of good practices concerning research accessibility. Ultimately, in ethical and accessible research dialogue is the basis.

**Dialog to podstawa. Doświadczenia osób Głuchych w Polsce z udziału w badaniach naukowych**

Pomimo dynamicznego rozwoju, nauka wciąż jest często niedostępna dla grup mniejszościowych, m.in. osób Głuchych korzystających z języka migowego. Społeczność Głuchych jest zróżnicowana pod względem tożsamości, preferencji językowych czy doświadczanej dyskryminacji. Pomimo to, naukowcy rzadko są zainteresowani doświadczeniami osób Głuchych z udziału w badaniach. Dlatego chcieliśmy poznać doświadczenia, opinie i potrzeby polskich osób Głuchych z udziału w badaniach naukowych. Chcieliśmy też włączyć perspektywę osób Głuchych z Polski w dyskusję naukową.

Przeprowadziliśmy badanie jakościowe - ankietę online z pytaniami umożliwiającymi otwarte, dłuższe odpowiedzi. Ankieta była dwujęzyczna – po polsku i w polskim języku migowym (PJM). Otrzymaliśmy 52 odpowiedzi. Były to osoby z różnych regionów Polski, w większości korzystające z PJM, o dobrej znajomości zarówno PJM, jak i języka pisanego. W zespole badawczym wszyscy jesteśmy słyszący, dlatego do współpracy przy analizie wyników zaprosiliśmy grupę osób Głuchych. Chcieliśmy wspólnie przedyskutować, jak rozumiemy i interpretujemy doświadczenia uczestników badania. Wykorzystaliśmy metodę analizy tematycznej w ujęciu Braun i Clarke. Ta metoda umożliwiła przedstawienie pogłębionego obrazu doświadczeń osób Głuchych. Wspólnie opracowaliśmy sześć tematów, przedstawionych w formie drogi, od historii, przez teraźniejszość, ku przyszłości.

Wyniki pokazują zainteresowanie osób Głuchych badaniami na tematy związane z głuchotą i społecznością Głuchych. Opisują obecną sytuację – niedostępność nauki: niedostosowane procedury, brak świadomości kulturowej naukowców, a także doświadczenie audyzmu – dyskryminacji ze strony słyszących naukowców. Sytuacja ta wywołuje negatywne uczucia, dyskomfort i rodzi nieufność do słyszących naukowców. Udział w badaniu jest wymagający psychicznie. Pomimo tych negatywnych doświadczeń, uczestnicy widzą naukę jako źródło nowych możliwości. Udział w badaniach przyczynia się do wspólnego rozwoju Głuchych i słyszących, w tym wzmocnienia społeczności Głuchych, walki z dyskryminacją i zwiększania świadomości społecznej. To daje Głuchym uczestnikom badań poczucie własnej wartości i satysfakcję z włączenia się w rozwój nauki i poprawę życia społecznego.

Uczestnicy podkreślali potrzebę zapewnienia dostępności - dwujęzycznych, materiałów przystosowanych do potrzeb różnorodnych Głuchych uczestników. Dostępności sprzyja też jasne wyjaśnienie celu, planowanych skutków badania oraz zapewnienie wynagrodzenia. Współpraca badawcza powinna być oparta na zaufaniu. Badacze powinni być obecni w społeczności Głuchych, a także świadomi jej norm i zwyczajów. To pozwala na zbudowanie relacji i otwartość w badaniu. W małej społeczności Głuchych ważna jest też dyskrecja i anonimowość. Uczestnicy wielokrotnie mówili o dostępnej i pełnej szacunku komunikacji, dlatego podsumowaliśmy analizę tematem: Dialog to podstawa.

Mamy nadzieję, że nasze badanie przyczyni się do poprawy dostępności i etyki badań naukowych i zainspiruje rozwój współpracy badawczej osób Głuchych i słyszących. Podstawą takiej współpracy są:

- dostępność całego procesu badawczego,
- świadomość kulturowa badaczy, a także
- aktywny udział osób Głuchych w nauce.

Współpraca powinna opierać się na dialogu, wzajemnym szacunku i zrozumieniu. To wymaga od naukowego świata krytycznego spojrzenia na swój stosunek do różnych grup społecznych, jak np. osoby Głuche. Wierzymy, że taki proces jest możliwy, jeśli obie strony będą otwarte na współpracę i refleksję nad własnymi przekonaniami. Zalecamy wsparcie projektów badawczych z udziałem lokalnej społeczności Głuchych oraz międzynarodową wymianę dobrych praktyk na temat dostępności. W końcu, w etycznych i dostępnych badaniach dialog to podstawa.
