# Supplementary material for: Dialogue matters. Exploring Deaf people’s research experiences in Poland
Source: J Deaf Stud Deaf Educ. 2026 Feb 23;31(3):460–70. doi: 10.1093/jdsade/enag006 (PMC13343195; doi:10.1093/jdsade/enag006)
Supplement: Survey-questions_enag006 [file survey-questions_enag006.docx]

**Supplementary file 2 – Open-ended questions**

(English translation)

The study consists of two parts. The first part (8 questions) concerns your experiences, needs, and opinions regarding research and its accessibility for d/Deaf people. There are no right or wrong answers. We are interested in your experiences and opinions, so we kindly ask you to answer the following questions honestly.

You may respond to questions 1–8 by recording a vlog (using a computer with a webcam or a phone), or by answering in writing.

The second part contains 8 short questions describing yourself.

1. Are you interested in science?
   - If so, please write which areas or topics interest you.
2. What comes to mind when you think of research?
   - If you have any experience with research (your own or that of your friends), please tell us about it.
3. What challenges or difficulties are associated with the participation of d/Deaf people in research?
   - For example: which challenges are the most significant? What difficulties are common to all participants? What difficulties are unique to d/Deaf people?
4. What should the research adapted for d/Deaf people look like?
   - For example: what would the study need to look like for you to feel comfortable participating?
5. What most encourages you to participate in research?
   - For example: what is most important when deciding whether to take part in a study? What accommodations or adaptations are present in research?
6. What most discourages you from participating in research?
   - For example: what might cause you to decline participation, even if the topic is interesting?
7. What kind of research is needed for d/Deaf people?
   - For example: in which areas? On what topics? Should scientific research be more accessible? Who should be involved in such studies?
8. Would you like to add anything else?
